# Pharmacogenetics of MicroRNAs and MicroRNAs Biogenesis Machinery in Pediatric Acute Lymphoblastic Leukemia

**DOI:** 10.1371/journal.pone.0091261

**Published:** 2014-03-10

**Authors:** Elixabet López-López, Ángela Gutiérrez-Camino, Maria Ángeles Piñán, José Sánchez-Toledo, Jose Javier Uriz, Javier Ballesteros, Purificación García-Miguel, Aurora Navajas, África García-Orad

**Affiliations:** 1 Department of Genetics, Physical Anthropology and Animal Physiology, Faculty of Medicine and Odontology, University of the Basque Country (UPV/EHU), Leioa, Spain; 2 Service of Hematology and Hemotherapy, University Hospital Cruces, Bilbao, Spain; 3 Service of Pediatric Oncology and Hematology, University Hospital Vall d' Hebron, VHIR, Barcelona, Spain; 4 Unit of Pediatric Oncohematology, University Hospital Donostia, San Sebastian, Spain; 5 Department of Neurosciences, Faculty of Medicine and Odontology, University of the Basque Country (UPV/EHU), Leioa, Spain; 6 Service of Pediatric Oncohematology, University Hospital La Paz, Madrid, Spain; 7 Unit of Pediatric Hematology/Oncology, University Hospital Cruces, Bilbao, Spain; B.C. Cancer Agency, Canada

## Abstract

Despite the clinical success of acute lymphoblastic leukemia (ALL) therapy, toxicity is frequent. Therefore, it would be useful to identify predictors of adverse effects. In the last years, several studies have investigated the relationship between genetic variation and treatment-related toxicity. However, most of these studies are focused in coding regions. Nowadays, it is known that regions that do not codify proteins, such as microRNAs (miRNAs), may have an important regulatory function. MiRNAs can regulate the expression of genes affecting drug response. In fact, the expression of some of those miRNAs has been associated with drug response. Genetic variations affecting miRNAs can modify their function, which may lead to drug sensitivity. The aim of this study was to detect new toxicity markers in pediatric B-ALL, studying miRNA-related polymorphisms, which can affect miRNA levels and function. We analyzed 118 SNPs in pre-miRNAs and miRNA processing genes in association with toxicity in 152 pediatric B-ALL patients all treated with the same protocol (LAL/SHOP). Among the results found, we detected for the first time an association between rs639174 in *DROSHA* and vomits that remained statistically significant after FDR correction. *DROSHA* had been associated with alterations in miRNAs expression, which could affect genes involved in drug transport. This suggests that miRNA-related SNPs could be a useful tool for toxicity prediction in pediatric B-ALL.

## Introduction

Acute lymphoblastic leukemia (ALL) is the most common childhood cancer, accounting for 30% of all pediatric malignancies [1]]. During the last decades, survival has been increased due to advances in chemotherapy for childhood ALL and cure rates now exceed 80% [2]]. However, despite the clinical success of therapy, patients often suffer from toxicity, requiring a dose reduction or cessation of treatment. Therefore, it would be very useful to identify predictors of these adverse effects [3]].

In the last years, several studies have investigated the relationship between genetic variation and treatment-related toxicity in ALL [4]–[10]. Nevertheless, most of these studies are focused in coding regions, which correspond only to about 1.5% of the entire genome.

Nowadays, it is known that regions that do not codify proteins have an important regulatory function. MiRNAs are small non-coding RNAs that regulate gene expression at the post-transcriptional level [11]. They are transcribed in the nucleus, as double stranded pri-miRNAs, which are processed to form the pre-miRNAs. The pre-miRNAs are exported to the cytoplasm and cleaved to produce two strands of miRNA [12], [13]. MiRNAs recognize their target mRNAs by binding to the 3′UTR of the target gene [14], which leads to an inhibition of translation or facilitated degradation of the target mRNA. All those steps are regulated by genes of the miRNA processing machinery.

MiRNAs can regulate genes involved in drug transport, metabolism and targets [15], affecting treatment response [16]. For example, upregulation of miR-125b, miR99a and miR-100 was related to resistance to vincristine and daunorubicin, and downregulation of miR-708 with resistance to glucocorticoids in pediatric B-ALL [17], [18]. This data indicate that changes in the expression or function of miRNAs could affect response to treatment.

Variations in miRNA expression and function may occur through genetic variations [12], [19]. Consequently, miRNA-related SNPs interfering with miRNA levels or function may lead to drug resistance or to drug sensitivity [20]. In fact, response to methotrexate (MTX), one of the most important drugs in ALL treatment, has been associated with SNP 829C>T near the miR-24 binding site in the 3′UTR of *DHFR*, that causes increased DHFR expression [21]. Also, in our group we observed that a polymorphism, that created a new miRNA binding site in *ABCC4* and could reduce ABCC4 expression, was associated with increased MTX plasma levels [22].

According to all those evidences, the aim of the present study was to determine if miRNA-related polymorphisms could be useful as new toxicity markers in pediatric B-ALL.

## Materials and Methods

### Ethics statement

University of the Basque Country (UPV/EHU) ethics committee board (CEISH) approval was obtained. Written Informed consent was obtained from all patients or their parents before sample collection.

### Patients

The patients included in this retrospective study were 152 children all diagnosed with B-ALL from 2000 to 2011 at the Pediatric Oncology Units of 4 Spanish hospitals (University Hospital Cruces; University Hospital Donostia; University Hospital Vall d'Hebrón and University Hospital La Paz).

### Treatment and toxicity evaluation

All patients were homogeneously treated with the LAL-SHOP 94/99 and 2005 protocols. The Induction phase consisted of treatment with daunorubicin, vincristine, prednisone, cyclophosphamide, asparaginase and triple intrathecal therapy. The consolidation phase consisted of high-dose methotrexate, mercaptopurine, cytarabine and triple intrathecal therapy [22].

Toxicity data were collected objectively, blinded to genotypes, from the patients' medical files. Toxicity was graded according to the Spanish Society of Pediatric Hematology and Oncology (SHOP) standards, adapted from the WHO criteria (grades 0–4). The highest grade of toxicity observed for each patient during the induction and consolidation therapy period was recorded.

### Genes and polymorphisms selection

We selected 21 genes in the pathway of miRNAs biogenesis and processing after literature review and using Patrocles database [23] (Table 1). In each gene, we covered all the SNPs with potentially functional effects using F-SNP, Fast-SNP, polymirTS [24], [25] and Patrocles [23] databases. We considered functional effects those causing amino acid changes, alternative splicing, in the promoter region in putative transcription factor binding sites, or disrupting/creating miRNAs targets. We also selected SNPs previously included in association studies in the literature. All SNPs were selected with a minor allele frequency greater than 5% (MAF≥0.05) in European/Caucasoid populations.

**Table 1:** Genes involved in miRNA biogenesis and processing.

COMPLEX	SUBGROUP	GENE
**DROSHA COMPLEX**	DGCR8	*DGCR8*
	DROSHA	*DROSHA*
	SMAD5	*SMAD5*
**NUCLEAR EXPORT COMPLEX**	XPO5	*XPO5*
	RAN	*RAN*
**DICER COMPLEX**	DICER1	*DICER1*
	TARBP2P	*TARBP2P*
**RISC COMPLEX**	Argonaute Family	*PIWIL1*
	(AGO)	*EIF2C1*
		*EIF2C2*
	TNRC	*TNRC6A*
		*TNRC6B*
	SND1	*SND1*
	GEMIN Complex	*DDX20*
		*GEMIN4*
		*GEMIN5*
	CCR4-NOT Complex	*CNOT1*
		*CNOT2*
		*CNOT3*
		*CNOT4*
		*CNOT6*

We searched for pre-miRNAs that had as putative targets genes involved in the pathways of the drugs used in LAL/SHOP protocol, using PharmGKB and mirWalk databases, and selected all the SNPs that had been described at the moment of the selection with a MAF>0.01 in European/Caucasic populations, using Patrocles and Ensembl databases and literature review.

### Genotyping

Genomic DNA was extracted with the phenol-chloroform method as previously described [8] from remission peripheral blood.

SNP genotyping was performed using TaqMan OpenArray Genotyping technology (Applied Biosystems, Life Technologies, Carlsbad, USA) according to the published Applied Biosystems protocol. The preliminary list of SNPs was filtered, using as criteria, suitability for the Taqman OpenArray platform.

Data were analyzed with Taqman Genotyper software for genotype clustering and calling. Duplicate samples were genotyped across the plates. In order to assess the Hardy-Weinberg equilibrium (HWE) status of each SNP, we genotyped in parallel 348 healthy adult individuals of Spanish origin.

### Statistical analysis

The χ2 or Fisher's exact test were used for HWE and toxicity analyses. The effect sizes of the associations were estimated by the odds ratios (OR's) from univariate logistic regression. The most significant test among dominant and recessive genetic models was selected. The results for each toxicity parameter were adjusted for multiple comparisons by the False Discovery Rate (FDR) [26]. In all cases the significance level was set at 5%. Analyses were performed by using R v2.11 software. Linkage disequilibrium analysis was performed with Haploview software v4.2.

## Results

### Patients' baseline characteristics

In this study, we have analyzed 152 B-ALL patients. Clinical data about MTX plasma concentration 72 h after infusion were available for 141 patients. Clinical data about other therapy-related toxicity in induction were available for 137 patients and in consolidation for 130 patients (Table 2).

**Table 2:** Characteristics of the study population.

No. of patients, n	152
Mean age at diagnosis ± SD, years	5.1456±3.41
Sex, n (%)	
Female	65 (42.76)
Male	87 (57.23)
Risk group, n (%)	
Standard	56 (40.57)
High	56 (40.57)
Very high	26 (18.84)
Treatment protocol, n (%)	
LAL-SHOP 94/99	65 (43.05)
LAL-SHOP 2005	86 (56.95)
MTX dose in consolidation, n (%)	
3 g/m^2^	73 (48.34)
5 g/m^2^	78 (51.66)
Toxicity during induction therapy, n (%)	
Any toxicity	79 (57.66)
Hepatic (Grade 2–4)	45 (32.84)
Vomits (Grade 2–4)	36 (26.28)
Diarrhea (Grade 2–4)	16 (11.67)
Mucositis (Grade 2–4)	29 (21.17)
Hyperbilirubinemia (Grade 1–4)	21 (15.32)
Renal (Grade 1–4)	5 (3.65)
Toxicity during consolidation therapy, n (%)	
Any toxicity	71 (54.61)
Hepatic (Grade 2–4)	39 (30)
Vomits (Grade 2–4)	31 (23.85)
Diarrhea (Grade 2–4)	9 (6.92)
Mucositis (Grade 2–4)	14 (10.77)
Hyperbilirubinemia (Grade 1–4)	11 (8.46)
Renal (Grade 1–4)	13 (10)
MTX concentration in plasma a, n (%)	
Higher than 0.2 uM at 72 h	51 (36.17)

a MTX levels were considered high if the concentration was over 0.2 µM at 72 h.

### Genotyping Results

We selected a total of 131 SNPs. After filtering for suitability for the Taqman Openarray platform, a final number of 118 SNPs (72 in 21 genes involved in miRNA biogenesis and 46 in 42 pre-miRNAs) was included in a Taqman Openarray Plate (Applied Biosystems) (Table S1 and S2).

A successful genotyping was obtained in 145 DNA samples (95.39%). In the genotyping process, 106 SNPs out of 118 were genotyped satisfactorily (89.83%). The failures were due to no PCR amplification, insufficient intensity for cluster separation, or poor or no cluster definition). The average genotyping rate for all SNPs was 97.81%. Of those 106 SNPs, 14 were not in HWE in a population of 348 healthy controls and were not considered for further analysis. In total, 26 SNPs were excluded from the association study (Table S3). The other 92 SNPs were used in the association studies.

### Analysis of the association with toxicity

In order to investigate if genetic variation may influence treatment toxicity, we tested the association between the 92 polymorphisms successfully genotyped that were in HWE in the control population and 15 different toxicity and pharmacokinetic parameters in the induction and consolidation phases (Table 2) (Table S4 and S5).

**Figure 1:**
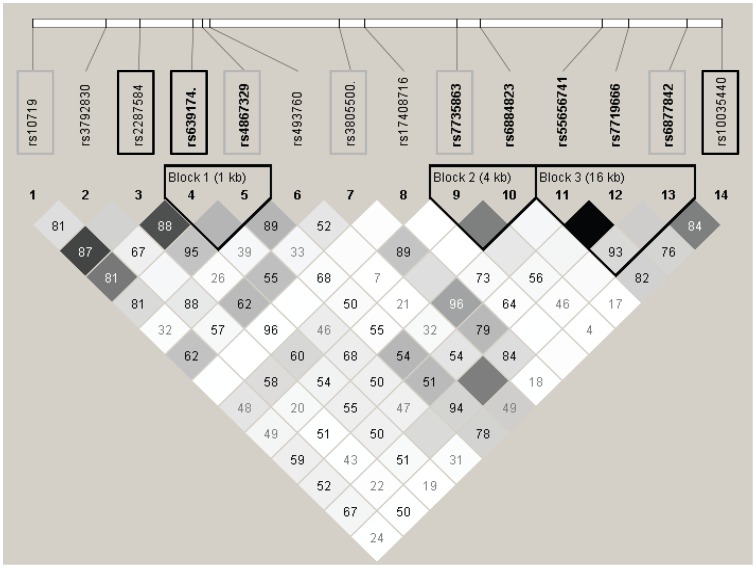
Linkage disequilibrium plot of the SNPs analyzed in DROSHA. White: r2 = 0, shades of grey: 0<r2<1, black: r2 = 1. Numbers in squares are D' values. Block definition is based on the Gabriel et al. method. The SNPs associated with toxicity are squared. Those among the top 10 associated SNPs are squared in black

In the genes of the miRNA biogenesis machinery, the most significant association was found between rs639174 in *DROSHA* and vomits in consolidation (p-value = 0.0003). This association remained statistically significant after FDR correction (p-corrected = 0.028). Interestingly, in *DROSHA* gene, a total of 8 SNPs out of 14 analyzed were associated with toxicity. Rs639174, rs2287584, rs10035440, rs4867329 and rs3805500 in *DROSHA* were among the top 10 associated SNPs in the biogenesis machinery (Table 3). Those SNPs are located along the whole gene and, in general, were not in high linkage disequilibrium among them (r^2^<0.8) (Figure 1).

**Table 3:** Most significant associations between polymorphisms in biogenesis machinery and toxicity parameters.

Gene	SNP	Toxicity	Phase	Genotype	No toxicity, n (%)	Toxicity, n (%)	OR (95% CI)	p-value	p-corrected
*DROSHA*	rs639174	Vomits	Cons	CC	47 (92.2)	4 (7.8)	1.00	0.0003	0.0276
				CT/TT	43 (65.2)	23 (34.9)	6.28 (2.01–19.64)		
	rs2287584	Vomits	Cons	TT	54 (88.5)	7 (11.5)	1.00	0.0026	0.1196
				CT/CC	41 (66.1)	21 (33.9)	3.95 (1.53–10.18)		
	rs10035440	Hyperbilirubinemia	Ind	TT	43 (72.9)	16 (27.1)	1.00	0.0041	0.1886
				CT/CC	65 (92.9)	5 (7.1)	0.23 (0.08–0.68)		
	rs4867329	Vomits	Cons	AA/AC	74 (72.6)	28 (27.5)	1.00	0.0088	0.2162
				CC	21 (95.5)	1 (4.6)	0.13 (0.02–0.98)		
	rs3805500	Hepatic toxicity	Cons	AA/AG	70 (68.0)	33 (32.0)	1.00	0.0091	0.2392
				GG	17 (94.4)	1 (5.6)	0.12 (0.02–0.98)		
*XPO5*	rs34324334	Hyperbilirubinemia	Ind	CC	101 (88.6)	13 (11.4)	1.00	0.0011	0.1012
				CT	7 (50.0)	7 (50.0)	7.77 (2.35–25.7)		
*TNRC6A*	rs6497759	Hepatic toxicity	Ind	GG	63 (76.8)	19 (23.2)	1.00	0.0013	0.1196
				AG/AA	23 (48.9)	24 (51.1)	3.46 (1.60–7.46)		
*TNRC6B*	rs9611280	Hepatic toxicity	Cons	GG	70 (67.3)	34 (32.7)	1.00	0.0042	0.2392
				AG	19 (95.0)	1 (5.0)	0.11 (0.01–0.84)		
*CNOT1*	rs11866002	Mucositis	Ind	CC/CT	98 (81.7)	22 (18.3)	1.00	0.0056	0.3956
				TT	4 (40.0)	6 (60.0)	6.68 (1.74–25.70)		
*DDX20*	rs197388	Renal toxicity	Ind	AA/AT	121 (97.6)	3 (2.4)	1.00	0.0068	0.4048
				TT	3 (60.0)	2 (40.0)	26.89 (3.21–225)		
*GEMIN4*	rs3744741	Hepatic toxicity	Ind	CC	70 (73.7)	25 (26.3)	1.00	0.0080	0.2392
				CT/TT	17 (48.6)	18 (51.4)	2.96 (1.33–6.63)		
*EIF2C1*	rs595961	Vomits	Ind	AA	67 (79.8)	17 (20.2)	1.00	0.0086	0.4017
				AG/GG	24 (57.1)	18 (42.9)	2.96 (1.31–6.65)		

Ind: induction. Cons: consolidation.

p-corrected =  p-value after FDR correction.

N.S.: non-significant, p-value>0.05 after FDR correction.

Among the pre-miRNAs, the most significant SNPs were rs12894467 in mir-300, associated with hepatic toxicity and hyperbilirubinemia in induction, and rs56103835 in mir-453, associated with vomits and MTX plasma levels in consolidation (Table 4).

**Table 4:** Most significant associations between polymorphisms in pre-miRNAs and toxicity parameters.

Gene	SNP	Toxicity	Phase	Genotype	No toxicity, n(%)	Toxicity, n(%)	OR (95% CI)	p-value	p-corrected
mir-300	rs12894467	Hepatic toxicity	Ind	CC/CT	82 (71.9)	32 (28.1)	1.00	0.0038	0.1748
				TT	5 (33.3)	10 (66.8)	5.12 (1.63–16.16)		
		Hyperbilirubinemia	Ind	CC/CT	99 (86.8)	15 (13.2)	1.00	0.0174	0.2990
				TT	9 (60.0)	6 (40.0)	4.40 (1.37–14.13)		
mir-449b	rs10061133	Renal toxicity	Ind	AA	111 (98.2)	2 (1.8)	1.00	0.0132	0.4048
				GA/GG	15 (83.3)	3 (16.7)	11.10 (1.71–71.9)		
mir-453	rs56103835	Vomits	Cons	AA	66 (83.5)	13 (16.5)	1.00	0.0141	0.2162
				GA/GG	28 (63.6)	16 (36.4)	2.90 (1.23–6.82)		
		MTX clearance	Cons	AA	62 (72.1)	24 (27.9)	1.00	0.0297	0.4554
				GA/GG	25 (53.2)	22 (46.8)	2.27 (1.08–4.77)		
mir-2053	rs10505168	Mucositis	Cons	TT	43 (82.7)	9 (17.3)	1.00	0.0154	0.4298
				CT/CC	68 (95.8)	3 (4.2)	0.21 (0.05–0.82)		
mir-423	rs6505162	Diarrhea	Ind	CC	25 (75.8)	8 (24.2)	1.00	0.0240	0.4618
				AC/AA	88 (91.7)	8 (8.3)	0.28 (0.10–0.83)		
mir-1307	rs7911488	Diarrhea	Ind	AA/AG	100 (90.1)	11 (9.9)	1.00	0.0241	0.4618
				GG	10 (66.7)	5 (33.3)	4.55 (1.31–15.72)		
mir-618	rs2682818	Hyperbilirubinemia	Ind	CC/AC	110 (85.4)	19 (14.7)	1.00	0.0247	0.3002
				AA	0 (0.0)	2 (100.0)	NE (NE- NE)		
mir-146a	rs2910164	Diarrhea	Ind	GG	70 (93.3)	5 (6.7)	1.00	0.0251	0.4618
				CG/CC	45 (80.4)	11 (19.6)	3.42 (1.11–10.50)		
mir-1206	rs2114358	Mucositis	Cons	AA/AG	96 (92.3)	8 (7.7)	1.00	0.0254	0.4298
				GG	15 (75.0)	5 (25.0)	4.57 (1.28–16.28)		
mir-577	rs34115976	Hyperbilirubinemia	Ind	CC	67 (78.8)	18 (21.2)	1.00	0.0261	0.3002
				CG/GG	41 (93.2)	3 (6.8)	0.27 (0.08–0.98)		
mir-604	rs2368393	Renal toxicity	Cons	AA	65 (95.6)	3 (4.4)	1.00	0.0271	0.6866
				AG/GG	47 (83.9)	9 (16.1)	4.15 (1.07–16.15)		
mir-492	rs2289030	Vomits	Ind	GG	90 (76.3)	28 (23.7)	1.00	0.0282	0.5188
				CG	6 (46.2)	7 (53.9)	3.75(1.16–12.08)		

Ind: induction. Cons: consolidation.

p-corrected = p-value after FDR correction.

N.S.: non-significant, p-value>0.05 after FDR correction.

## Discussion

It is known that ALL treatment can cause toxicity and toxicity predictors are needed. It has been proposed that miRNA-related SNPs interfering with miRNA function may lead to drug resistance or to drug sensitivity [20]. However, there are very few studies analyzing the role of polymorphisms in miRNA biogenesis genes and in miRNAs and none of them had been performed in pediatric ALL.

In this study, it is worth noting that we have found for the first time an association between rs639174 in *DROSHA* and vomits and this association remained statistically significant after FDR correction. In *DROSHA*, rs639174 is an intronic SNP with a putative role in transcriptional regulation (TR). This SNP had been previously associated with head and neck cancer recurrence, suggesting that in some way this SNP may have a functional effect on the gene [27]. Interestingly, other 7 polymorphisms in *DROSHA* with a putative role in splicing and transcriptional regulation were associated with toxicity in induction and consolidation (rs10035440, rs2287584, rs4867329, rs3805500, rs6877842, rs10719 and rs7735863), although they did not remain statistically significant after FDR correction. The SNP rs3805500, which is associated with hepatic toxicity and vomits in our study, is in LD with the SNP rs640831, previously associated with reduced *DROSHA* mRNA expression and with expression changes in 56 miRNAs out of 199 analyzed [28]. This can be understood knowing that *DROSHA* (*RNASEN*) encodes an RNAse III enzyme, involved in pri-miRNAs maturation into pre-miRNAs [29]. This general alteration of miRNA expression could lead to changes in the expression of genes involved in response to treatment, which could explain the effect we have observed on toxicity during pediatric ALL treatment.

As far as we know, this is the first time that polymorphisms in miRNA processing genes have been associated with toxicity after treatment in cancer patients. Knowing that literature about the function of these genes and their implication in pharmacogenetics is scarce, our results indicate that these genes and polymorphisms could be of relevance in the study of drug response.

We also found associations between SNPs in pre-miRNAs and toxicity. Interestingly, SNPs associated with toxicity in induction were different from those associated with toxicity in consolidation, in which different drugs are given. This may mean that each miRNA regulates specific drug pathways. Although these associations did not remain significant after FDR correction and are currently of uncertain significance, we still consider that it is interesting to discuss them due to their putative roles in the regulation of drug pathways.

The most significant association between SNPs in pre-miRNAs and toxicity in induction was with rs12894467 in the premature mir-300, which could affect the structure and processing of this miRNA. Interestingly, among the predicted targets of mir-300, we can find the transporters *ABCC1* and *ABCB1*, with a role in vincristine detoxification, and the enzyme *ALDH5A1*, involved in cyclophosphamide inactivation. If the rs12894467 T risk allele caused an upregulation of mir-300, this could explain a downregulation of its targets, leading to an increased effect of the drugs used in the induction phase.

The most significant association in consolidation was between the SNP rs56103835 in the premature mir-453 (also known as mir-323b-5p) and both MTX plasma levels and vomits. This miRNA has as putative target genes *ABCC1*, *ABCB1*, *ABCC2* and *ABCC4*, which are involved in MTX transport. The SNP rs56103835, in which G allele is associated with higher risk of toxicity, is in the pre-miRNA, and thus could influence miRNA biogenesis and levels of mature mir-453. If mir-453 is up-regulated, it would decrease the activity of *ABCC1*, *ABCB1*, *ABCC2* and *ABCC4* genes, and the higher MTX plasma levels and toxicity observed could be explained. In fact, in a previous study carried out by our group, we showed the relevance of genetic variation in *ABCC2* and *ABCC4* genes for MTX toxicity [22].

In conclusion, we have found for the first time an association between rs639174 in DROSHA and vomits and other more uncertain associations between polymorphism in genes involved in miRNAs biogenesis and in pre-miRNAs and toxicity during pediatric ALL treatment. These results suggest that miRNA-related SNPs, which can be important in drug pharmacokinetics and dynamics, could be useful as toxicity markers in pediatric ALL. We open a new promising field of investigation, involving the study of miRNA-related polymorphisms in pediatric ALL treatment. Further studies are needed in order to assess the relevance of these SNPs in ALL pharmacogenetics.

## Supporting Information

Table S1 **Single Nucleotide Polymorphisms selected in microRNA processing genes and selection criteria.**
(PDF)

Table S2 **Characteristics of the Single Nucleotide Polymorphisms in microRNAs.**
(PDF)

Table S3 **SNPs excluded from the miRNAs pathway association study.**
(PDF)

Table S4 **Full list of significant associations between polymorphisms in biogenesis machinery and toxicity parameters.**
(PDF)

Table S5 **Full list of significant associations between polymorphisms in pre-miRNAs and toxicity parameters.**
(PDF)
