# Supplementary material for: Pharmacogenetics of MicroRNAs and MicroRNAs Biogenesis Machinery in Pediatric Acute Lymphoblastic Leukemia
Source: PLoS One. 2014 Mar 10;9(3):e91261. doi: 10.1371/journal.pone.0091261 (PMC3948785; doi:10.1371/journal.pone.0091261)
Supplement: Table S1 — Single Nucleotide Polymorphisms selected in microRNA processing genes and selection criteria. (PDF) [file pone.0091261.s001.pdf]

Table S1. Single Nucleotide Polymorphisms selected in microRNA processing genes and selection criteria

Gene	SNP ID	Alleles	Chr	Location	Function	Reason for selection
CNOT1	rs11644694	G>A	16	58557342	non-synonymous	NS, SR
	rs11866002	C>T	16	58587737	synonymous	SR
	rs37060	C>T	16	58566304	intronic	SR
CNOT2	rs10506586	C>A	12	70715490	non-synonymous	NS, SR
CNOT3	rs42318	G>A	19	54657069	non-synonymous	NS
CNOT4	rs1003226	T>C	7	135046552	3'UTR	SR
	rs3763425	C>T	7	135195320	upstream	UR
	rs3812265	C>T	7	135048804	non-synonymous	NS, SR
CNOT6	rs11738060	T>A	5	180004154	3'UTR	MIRTS
	rs6877400	T>C	5	179996111	synonymous	SR
DGCR8	rs1640299	T>G	22	20098359	3'UTR	BIB
	rs35987994	T>C	22	20074006	non-synonymous	NS
	rs3757	G>A	22	20099331	3'UTR	MIRTS, BIB
	rs417309	G>A	22	20098544	3'UTR	3UTR BIB
	rs9606248	A>G	22	20087539	intronic	BIB
DICER1	rs1057035	T>C	14	95554142	3'UTR	MIRTS
	rs1209904	C>T	14	95563712	intronic	BIB
	rs13078	T>A	14	95556747	3'UTR	3UTR, BIB
	rs3742330	A>G	14	95553362	3'UTR	BIB
DROSHA	rs10035440	T>C	5	31539463	upstream	BIB
	rs10719	C>T	5	31401447	3'UTR	3UTR, BIB
	rs17408716	A>G	5	31467952	intronic	BIB
	rs2287584	T>C	5	31423007	synonymous	SR, BIB
	rs3792830	T>C	5	31416248	intronic	BIB
	rs3805500	T>C	5	31462977	intronic	BIB
	rs4867329	A>C	5	31435627	intronic	BIB
	rs493760	T>C	5	31437040	intronic	BIB
	rs55656741	G>A	5	31515657	non-synonymous	NS, SR
	rs639174	C>T	5	31433647	intronic	BIB
	rs6877842	G>C	5	31532638	upstream	BIB
	rs6884823	G>A	5	31491121	intronic	BIB
	rs7719666	C>T	5	31520778	intronic	BIB
	rs7735863	G>A	5	31486540	intronic	BIB
EIF2C1	rs595961	A>G	1	36367780	intronic	BIB
	rs636832	G>A	1	36363475	intronic	BIB
EIF2C2	rs2292778	C>T	1	141568622	synonymous	SR
	rs2293939	G>A	1	141551407	synonymous	SR
	rs4961280	C>A	1	141647414	upstream	UR, BIB
DDX20	rs197388	T>A	1	112297482	upstream	UR, BIB
	rs197412	T>C	1	112308953	non-synonymous	NS, BIB
	rs197414	C>A	1	112309123	non-synonymous	NS, BIB
	rs563002	T>C	1	112317135	downstream	BIB
GEMIN4	rs1062923	T>C	17	649067	non-synonymous	NS, BIB
	rs2740348	G>C	17	649935	non-synonymous	NS, BIB
	rs34610323	C>T	17	648546	non-synonymous	NS
	rs3744741	C>T	17	649232	non-synonymous	NS, BIB
	rs7813	C>T	17	648186	non-synonymous	NS, BIB
	rs910924	C>T	17	655920	5'UTR	5UTR, BIB
GEMIN5	rs1974777	A>G	5	154291409	non-synonymous	NS
	rs6865950	G>A	5	154275786	non-synonymous	NS
	rs816736	T>C	5	154271948	synonymous	SR
PIWIL1	rs1106042	G>A	12	130841638	non-synonymous	NS, SR, BIB
RAN	rs11061209	G>A	12	131364988	downstream	BIB
	rs14035	C>T	12	131361241	3'UTR	MIRTS, BIB
SMAD5	rs3764941	A>C	5	135469527	non-synonymous	NS, SR
	rs3764942	G>A	5	135469500	intronic	SR
SND1	rs17151639	A>G	7	127637816	non-synonymous	NS
	rs17676986	C>T	7	127636958	intronic	TR
	rs322825	C>T	7	127721507	synonymous	SR
	rs3823994	T>A	7	127669857	intronic	SR
TNRC6A	rs6497759	G>A	16	24801737	non-synonymous	NS
TNRC6B	rs139919	T>C	22	40726183	3'UTR	MIRTS
	rs2413621	T>C	22	40673999	intronic	SR
	rs470113	A>G	22	40729614	3'UTR	MIRTS
	rs4821943	A>G	22	40722745	3'UTR	MIRTS
	rs9611280	G>A	22	40552119	non-synonymous	NS, SR
TARBP2P	rs784567	C>T	12	53894465	upstream	BIB
XPO5	rs1106841	A>C	6	43496662	synonymous	SR
	rs2227301	G>A	6	43485283	downstream	BIB
	rs2257082	C>T	6	43492578	synonymous	SR, BIB
	rs34324334	C>T	6	43535018	non-synonymous	NS, SR
	rs7755135	C>T	6	43490809	3'UTR	MIRTS

3UTR: 3'UTR regulation; 5UTR: 5'UTR regulation; BIB: Bibliographic; MIRTS: miRNA target site; NS: non-synonymous; SR: Splicing regulation; UR: Upstream regulation.
