# Supplementary material for: Pharmacogenetics of MicroRNAs and MicroRNAs Biogenesis Machinery in Pediatric Acute Lymphoblastic Leukemia
Source: PLoS One. 2014 Mar 10;9(3):e91261. doi: 10.1371/journal.pone.0091261 (PMC3948785; doi:10.1371/journal.pone.0091261)
Supplement: Table S2 — Characteristics of the Single Nucleotide Polymorphisms in microRNAs. (PDF) [file pone.0091261.s002.pdf]

Table S2. Characteristics of the Single Nucleotide Polymorphisms in microRNAs

**Gene**	**SNP ID**	**Alleles**	**Chr**	**Location**	**Position**
mir-106b	rs72631827	G>T	7	99691652	premature
mir-1178	rs7311975	T>C	12	120151493	premature
mir-1206	rs2114358	T>C	8	129021179	premature
mir-1255b-1	rs6841938	G>A	4	36428048	mature
mir-1265	rs11259096	T>C	10	14478618	premature
mir-1269	rs73239138	G>A	4	67142620	mature
mir-1274a	rs318039	C>T	5	41475766	premature
mir-1282	rs11269	G>T	15	44085909	premature
mir-1294	rs13186787	A>G	5	153726769	premature
mir-1302-4	rs10173558	T>C	2	208133995	premiRNA flanking region
mir-1307	rs7911488	A>G	10	105154089	premature
mir-146a	rs2910164	G>C	5	159912418	premature
mir-149	rs2292832	C>T	2	241395503	premature
mir-154	rs41286570	G>A	14	101526127	mature
mir-16-1	rs72631826	T>C	13	50623143	premature
mir-1908	rs174561	T>C	11	61582708	premature
Mir-196a-2	rs11614913	C>T	12	54385599	premature
mir-2053	rs10505168	A>G	8	113655752	premature
mir-2110	rs17091403	C>T	10	115933905	premature
mir-216a	rs41291179	A>T	2	56216090	premature
mir-220a	rs72631817	T>C	X	122696014	premature
mir-222	rs72631825	G>A	X	45606471	premature
mir-27a	rs895819	T>C	19	13947292	premature
mir-300	rs12894467	C>T	14	101507727	premature
mir-423	rs6505162	A>C	17	28444183	premature
mir-449b	rs10061133	A>G	5	54466544	mature
mir-453	rs56103835	T>C	14	101522556	premature
mir-492	rs2289030	C>G	12	95228286	premature
mir-499	rs3746444	T>C	20	33578251	seed
mir-548a-1	rs12197631	T>G	6	18572056	premature
mir-548h-3	rs9913045	G>A	12	13446924	mature
mir-548h-4	rs73235382	A>T	8	26906437	premature
mir-577	rs34115976	C>G	4	115577997	premature
mir-585	rs62376934	G>A	5	168690612	premature
mir-595	rs4909237	C>T	7	158325503	premature
mir-603	rs11014002	C>T	10	24564653	premature
mir-604	rs2368392	C>T	10	29834003	premature
mir-604	rs2368393	T>C	10	29833998	premature
mir-605	rs2043556	A>G	10	53059406	premature
mir-608	rs4919510	C>G	10	102734778	mature
mir-612	rs12803915	G>A	11	65211979	premature
mir-612	rs550894	G>T	11	65211940	premature
mir-618	rs2682818	C>A	12	81329536	premature
mir-624	rs11156654	T>A	14	31483955	premiRNA flanking region
mir-656	rs58834075	C>T	14	101533093	premature
mir-943	rs1077020	T>C	4	1988193	premature
