# Supplementary material for: Pharmacogenetics of MicroRNAs and MicroRNAs Biogenesis Machinery in Pediatric Acute Lymphoblastic Leukemia
Source: PLoS One. 2014 Mar 10;9(3):e91261. doi: 10.1371/journal.pone.0091261 (PMC3948785; doi:10.1371/journal.pone.0091261)
Supplement: Table S3 — SNPs excluded from the miRNAs pathway association study. (PDF) [file pone.0091261.s003.pdf]

Table S3. SNPs excluded from the miRNAs pathway association study.

SNP ID	Gene	Alleles	Reason for exclusion
rs1003226	*CNOT4*	T>C	Genotyping failure
rs11738060	*CNOT6*	T>A	Genotyping failure
rs34610323	*GEMIN4*	C>T	Genotyping failure
rs73239138	mir-1269	G>A	Genotyping failure
rs318039	mir-1274a	C>T	Genotyping failure
rs72631826	mir-16-1	T>C	Genotyping failure
rs72631825	mir-222	G>A	Genotyping failure
rs12197631	mir-548a-1	T>G	Genotyping failure
rs11014002	mir-603	C>T	Genotyping failure
rs2368392	mir-604	C>T	Genotyping failure
rs11061209	*RAN*	G>A	Genotyping failure
rs493760	*DROSHA*	T>C	Genotyping failure
rs42318	*CNOT3*	G>A	No HWE
rs3757	*DGCR8*	G>A	No HWE
rs3742330	*DICER1*	A>G	No HWE
rs7813	*GEMIN4*	C>T	No HWE
rs910924	*GEMIN4*	C>T	No HWE
rs816736	*GEMIN5*	T>C	No HWE
rs2292832	mir-149	C>T	No HWE
rs174561	mir-1908	T>C	No HWE
rs4919510	*mir-608*	C>G	No HWE
rs11156654	mir-624	T>A	No HWE
rs55656741	*DROSHA*	G>A	No HWE
rs7719666	*DROSHA*	C>T	No HWE
rs2413621	*TNRC6B*	T>C	No HWE
rs470113	*TNRC6B*	A>G	No HWE
