# Supplementary material for: Pharmacogenetics of MicroRNAs and MicroRNAs Biogenesis Machinery in Pediatric Acute Lymphoblastic Leukemia
Source: PLoS One. 2014 Mar 10;9(3):e91261. doi: 10.1371/journal.pone.0091261 (PMC3948785; doi:10.1371/journal.pone.0091261)
Supplement: Table S4 — Full list of significant associations between polymorphisms in biogenesis machinery and toxicity parameters. (PDF) [file pone.0091261.s004.pdf]

Table S4. Full list of significant associations between polymorphisms in biogenesis machinery and toxicity parameters.

Gene	SNP	Toxicity	Phase	Genotype	No toxicity n (%)	Toxicity n (%)	OR (95% CI)	p-value
DROSHA	rs639174	Vomits	Cons	CC	47 (92.2)	4 (7.8)	1.00	0.0003*
				CT/TT	43 (65.2)	23 (34.9)	6.28 (2.01-19.64)	
	rs2287584	Vomits	Cons	TT	54 (88.5)	7 (11.5)	1.00	0.0026
				CT/CC	41 (66.1)	21 (33.9)	3.95 (1.53-10.18)	
		Vomits	Ind	TT	41 (65.1)	22 (34.9)	1.00	0.0455
				CT/CC	54 (80.6)	13 (19.4)	0.45 (0.20-1.00)	
	rs10035440	Hyperbilirubinemia	Ind	TT	43 (72.9)	16 (27.1)	1.00	0.0041
				CT/CC	65 (92.9)	5 (7.1)	0.23 (0.08-0.68)	
		Diarrhea	Cons	TT/CT	104 (94.6)	6 (5.5)	1.00	0.0405
				CC	9 (75.0)	3 (25.0)	5.78 (1.23-27.06)	
	rs4867329	Vomits	Cons	AA/AC	74 (72.6)	28 (27.5)	1.00	0.0088
				CC	21 (95.5)	1 (4.6)	0.13 (0.02-0.98)	
	rs3805500	Hepatic toxicity	Cons	AA/AG	70 (68.0)	33 (32.0)	1.00	0.0091
				GG	17 (94.4)	1 (5.6)	0.12 (0.02-0.98)	
		Vomits	Cons	AA	41 (89.1)	5 (10.9)	1.00	0.0137
				AG/GG	53 (70.7)	22 (29.3)	3.40 (1.19-9.76)	
	rs10719	Vomits	Cons	GG	56 (83.6)	11 (16.4)	1.00	0.0259
				AG/AA	35 (66.0)	18 (34.0)	2.62 (1.11-6.19)	
		MTX plasma levels	Cons	GG/AG	77 (63.6)	44 (36.4)	1.00	0.0279
				AA	9 (100)	0 (0.0)	NE (NE-NE)	
	rs7735863	Hepatic toxicity	Cons	GG	58 (66.7)	29 (33.3)	1.00	0.0266
				AG/AA	30 (85.7)	5 (14.3)	0.33 (0.12-0.95)	
	rs6877842	Diarrhea	Ind	GG	73 (92.4)	6 (7.6)	1.00	0.0359
				GC/CC	39 (79.6)	10 (20.4)	3.12 (1.05-9.23)	
		Hepatic toxicity	Cons	GG	59 (77.6)	17 (22.4)	1.00	0.0405
				CG/CC	27 (60.0)	18 (40.0)	2.31 (1.04-5.17)	
XPO5	rs34324334	Hyperbilirubinemia	Ind	CC	101 (88.6)	13 (11.4)	1.00	0.0011
				CT	7 (50.0)	7 (50.0)	7.77 (2.35-25.7)	
		Hepatic toxicity	Cons	CC	73 (68.2)	34 (31.8)	1.00	0.0101
				CT	14 (100)	0 (0.0)	NE (NE-NE)	
	rs7755135	Mucositis	Ind	GG	68 (73.9)	24 (26.1)	1.00	0.0251
				AG/AA	33 (89.2)	4 (10.8)	2.68 (1.14-6.32)	
TNRC6A	rs6497759	Hepatic toxicity	Ind	GG	63 (76.8)	19 (23.2)	1.00	0.0013
				AG/AA	23 (48.9)	24 (51.1)	3.46 (1.60-7.46)	
TNRC6B	rs9611280	Hepatic toxicity	Cons	GG	70 (67.3)	34 (32.7)	1.00	0.0042
				AG	19 (95.0)	1 (5.0)	0.11 (0.01-0.84)	
	rs139919	Mucositis	Cons	TT	73 (86.9)	11 (13.1)	1.00	0.0327
				CT/CC	36 (100)	0 (0.0)	NE (NE-NE)	
CNOT1	rs11866002	Mucositis	Ind	CC/CT	98 (81.7)	22 (18.3)	1.00	0.0056
				TT	4 (40.0)	6 (60.0)	6.68 (1.74-25.70)	
		Hepatic toxicity	Cons	CC	38 (63.3)	22 (36.7)	1.00	0.0480
				CT/TT	50 (79.4)	13 (20.6)	0.45 (0.20-1.00)	
	rs37060	Hepatic toxicity	Ind	GG	47 (77.1)	14 (22.9)	1.00	0.0152
				AG/AA	40 (57.1)	30 (42.9)	2.52 (1.18-5.39)	
		Hyperbilirubinemia	Ind	GG	56 (91.8)	5 (8.2)	1.00	0.0193
				AG/AA	54 (77.1)	16 (22.9)	3.32 (1.14-9.69)	
DDX20	rs197388	Renal toxicity	Ind	AA/AT	121 (97.6)	3 (2.4)	1.00	0.0068
				TT	3 (60.0)	2 (40.0)	26.89 (3.21-225)	
	rs563002	Hyperbilirubinemia	Cons	TT	73 (96.1)	3 (3.9)	1.00	0.0252
				CT/CC	37 (84.1)	7 (15.9)	4.60 (1.12-18.84)	
GEMIN4	rs3744741	Hepatic toxicity	Ind	CC	70 (73.7)	25 (26.3)	1.00	0.0080
				CT/TT	17 (48.6)	18 (51.4)	2.96 (1.33-6.63)	
	rs1062923	MTX clearance	Cons	AA	73 (71.6)	29 (28.4)	1.00	0.0191
				AG/GG	15 (48.4)	16 (51.6)	2.69 (1.18-6.13)	
		Vomits	Cons	AA	74 (81.3)	17 (18.7)	1.00	0.0366
				AG/GG	20 (62.5)	12 (37.5)	2.61 (1.07-6.35)	
EIF2C1	rs595961	Vomits	Ind	AA	67 (79.8)	17 (20.2)	1.00	0.0086
				AG/GG	24 (57.1)	18 (42.9)	2.96 (1.31-6.65)	
		Mucositis	Cons	AA	70 (87.5)	10 (12.5)	1.00	0.0294
				AG/GG	39 (100)	0 (0.0)	NE (NE-NE)	
CNOT4	rs3812265	Mucositis	Ind	CC	61 (72.6)	23 (27.4)	1.00	0.0129
				CT/TT	39 (90.7)	4 (9.3)	0.27 (0.09-0.85)	
		Mucositis	Cons	CC	74 (94.9)	4 (5.1)	1.00	0.0183
				CT/TT	34 (81.0)	8 (19.1)	4.35 (1.23-15.5)	

Table S4 continuation. Full list of significant associations between polymorphisms in biogenesis machinery and toxicity parameters.

Gene	SNP	Toxicity	Phase	Genotype	No toxicity n (%)	Toxicity n (%)	OR (95% CI)	p
TARBP2P	rs784567	Vomits	Ind	GG/AG	66 (68.8)	30 (31.3)	1.00	0.0131
				AA	27 (90.0)	3 (10.0)	0.24 (0.07-0.87)	
DICER1	rs1209904	Diarrhea	Cons	CC	58 (98.3)	1 (1.7)	1.00	0.0141
				CT/TT	56 (87.5)	8 (12.5)	8.29 (1.00-68.39)	
	rs13078	Diarrhea	Cons	TT	68 (97.1)	2 (2.9)	1.00	0.0255
				AT/AA	45 (86.5)	7 (13.8)	5.29 (1.05-26.62)	
		MTX clearance	Cons	TT	57 (72.2)	22 (27.9)	1.00	0.0400
				AT/AA	29 (54.7)	24 (45.3)	2.14 (1.03-4.45)	
SND1	rs3823994	MTX clearance	Cons	AA/AT	84 (68.9)	38 (31.2)	1.00	0.0165
				TT	4 (33.3)	8 (66.7)	4.42 (1.25-15.58)	
	rs322825	Mucositis	Ind	CC	39 (69.6)	17 (30.4)	1.00	0.0394
				CT/TT	62 (84.9)	11 (15.1)	0.41 (0.17-0.96)	
DGCR8	rs417309	Renal toxicity	Ind	GG/AG	125 (96.9)	4 (3.1)	1.00	0.0346
				AA	0 (0.0)	1 (100)	NE (NE-NE)	

Ind: induction. Cons: consolidation

*p-value<0.05 after FDR correction
