# Supplementary material for: Pharmacogenetics of MicroRNAs and MicroRNAs Biogenesis Machinery in Pediatric Acute Lymphoblastic Leukemia
Source: PLoS One. 2014 Mar 10;9(3):e91261. doi: 10.1371/journal.pone.0091261 (PMC3948785; doi:10.1371/journal.pone.0091261)
Supplement: Table S5 — Full list of significant associations between polymorphisms in pre-miRNAs and toxicity parameters. (PDF) [file pone.0091261.s005.pdf]

Table S5. Full list of significant associations between polymorphisms in pre-miRNAs and toxicity parameters.

Gene	SNP	Toxicity	Phase	Genotype	No toxicity n(%)	Toxicity n(%)	OR (95% CI)	p-value
mir-300	rs12894467	Hepatic toxicity	Ind	CC/CT	82 (71.9)	32 (28.1)	1.00	0.0038
				TT	5 (33.3)	10 (66.8)	5.12 (1.63-16.16)	
		Hyperbilirubinemia	Ind	CC/CT	99 (86.8)	15 (13.2)	1.00	0.0174
				TT	9 (60.0)	6 (40.0)	4.40 (1.37-14.13)	
mir-449b	rs10061133	Renal toxicity	Ind	AA	111 (98.2)	2 (1.8)	1.00	0.0132
				GA/GG	15 (83.3)	3 (16.7)	11.10 (1.71-71.9)	
mir-453	rs56103835	Vomits	Cons	AA	66 (83.5)	13 (16.5)	1.00	0.0141
				GA/GG	28 (63.6)	16 (36.4)	2.90 (1.23-6.82)	
		MTX clearance	Cons	AA	62 (72.1)	24 (27.9)	1.00	0.0297
				GA/GG	25 (53.2)	22 (46.8)	2.27 (1.08-4.77)	
mir-2053	rs10505168	Mucositis	Cons	TT	43 (82.7)	9 (17.3)	1.00	0.0154
				CT/CC	68 (95.8)	3 (4.2)	0.21 (0.05-0.82)	
mir-423	rs6505162	Diarrhea	Ind	CC	25 (75.8)	8 (24.2)	1.00	0.0240
				AC/AA	88 (91.7)	8 (8.3)	0.28 (0.10-0.83)	
mir-1307	rs7911488	Diarrhea	Ind	AA/AG	100 (90.1)	11 (9.9)	1.00	0.0241
				GG	10 (66.7)	5 (33.3)	4.55 (1.31-15.72)	
		Mucositis	Ind	AA	44 (88.0)	6 (12.0)	1.00	0.0311
				AG/GG	55 (72.4)	21 (27.6)	2.80 (1.04-7.54)	
mir-618	rs2682818	Hyperbilirubinemia	Ind	CC/AC	110 (85.4)	19 (14.7)	1.00	0.0247
				AA	0 (0.0)	2 (100.0)	NE (NE- NE)	
mir-146a	rs2910164	Diarrhea	Ind	GG	70 (93.3)	5 (6.7)	1.00	0.0251
				CG/CC	45 (80.4)	11 (19.6)	3.42 (1.11-10.50)	
		Mucositis	Ind	GG	64 (85.3)	11 (14.7)	1.00	0.0309
				CG/CC	39 (69.6)	17 (30.4)	2.54 (1.08-5.97)	
mir-1206	rs2114358	Mucositis	Cons	AA/AG	96 (92.3)	8 (7.7)	1.00	0.0254
				GG	15 (75.0)	5 (25.0)	4.57 (1.28-16.28)	
		Diarrhea	Cons	AA/AG	98 (95.2)	5 (4.9)	1.00	0.0365
				GG	16 (80.0)	4 (20.0)	4.90 (1.49-20.21)	
mir-577	rs34115976	Hyperbilirubinemia	Ind	CC	67 (78.8)	18 (21.2)	1.00	0.0261
				CG/GG	41 (93.2)	3 (6.8)	0.27 (0.08-0.98)	
mir-604	rs2368393	Renal toxicity	Cons	AA	65 (95.6)	3 (4.4)	1.00	0.0271
				AG/GG	47 (83.9)	9 (16.1)	4.15 (1.07-16.15)	
mir-492	rs2289030	Vomits	Ind	GG	90 (76.3)	28 (23.7)	1.00	0.0282
				CG	6 (46.2)	7 (53.9)	3.75(1.16-12.08)	
mir-27a	rs895819	Hyperbilirubinemia	Ind	TT	46 (92.0)	4 (8.0)	1.00	0.0320
				CT/CC	61 (78.2)	17 (21.8)	3.20 (1.01-10.17)	
mir-196a-2	rs11614913	Diarrhea	Ind	CC/CT	101 (90.2)	11 (9.8)	1.00	0.0407
				TT	12 (70.6)	5 (29.4)	3.83 (1.14-12.89)	
mir-1294	rs13186787	Hyperbilirubinemia	Cons	AA	110 (93.2)	8 (6.8)	1.00	0.0424
				AG	3 (60.0)	2 (40.0)	9.17 (1.33-63.01)	
mir-656	rs58834075	Hyperbilirubinemia	Ind	GG	105 (86.1)	17 (13.9)	1.00	0.0426
				GA	3 (50.0)	3 (50.0)	6.18(1.15-33.15)	
mir-2110	rs17091403	Vomits	Cons	CC	84 (80.0)	21 (20.0)	1.00	0.0471
				CT	11 (57.9)	8 (42.1)	2.91 (1.04-8.14)	

Ind: induction. Cons: consolidation
